# Epidemiological characteristics of alcohol-related liver disease in China: a systematic review and meta-analysis

**DOI:** 10.1186/s12889-023-15645-4

**Published:** 2023-07-01

**Authors:** Zongzhe Tang, Yajie Ding, Wei Zhang, Ru Zhang, Liuxin Zhang, Minxian Wang, Min Wang, Yue Chen, Jie Wang

**Affiliations:** 1 Department of Fundamental and Community Nursing, School of Nursing, Nanjing Medical University, 101 Longmian Avenue, Nanjing, 211166 Jiangsu China; 2 Shanghai Cancer Institute, Shanghai, 200032 China; 3 The Nethersole School of Nursing, Faculty of Medicine, The Chinese University of Hong Kong, Sha Tin, New Territories, Hong Kong (SAR), China; 4 Hospital Development Management Office, Nanjing Medical University, Nanjing, China

**Keywords:** Alcohol-related liver disease, Prevalence, China, Meta-analysis, Systematic review

## Abstract

**Background:**

This meta-analysis aimed to explore the epidemiological characteristics of alcohol-related liver disease (ALD) in China.

**Methods:**

Studies published between January 2000 and January 2023 were searched from 3 databases in English and 3 databases in Chinese. DerSimonian-Laird’s random-effects model was adopted to calculate the pooled prevalence.

**Results:**

A total of 21 studies were included. The pooled prevalence of ALD was 4.8% (95% CI, 3.6%-6.2%) in the general population, 9.3% (95% CI, 4.4%-16.0%) in males, and 2.0% (95% CI, 0.0%-6.7%) in females. The prevalence was the highest in western China (5.0% [95% CI, 3.3%-6.9%]) and the lowest in central China (4.4% [95% CI, 4.0%-4.8%]). The prevalence among people with different drinking histories (less than 5 years, 5 to 10 years, and over 10 years) was 0.9% (95% CI, 0.2%-1.9%), 4.6% (95% CI, 3.0%-6.5%), and 9.9% (95% CI, 6.5%-14.0%), respectively. The prevalence in 1999–2004 was 4.7% (95% CI, 3.0%-6.7%) and then changed from 4.3% (95% CI, 3.5%-5.3%) in 2005–2010 to 6.7% (95% CI, 5.3%-8.3%) in 2011–2016.

**Conclusions:**

The prevalence of ALD in China has increased in recent decades, with population-related variations. Targeted public health strategies are needed, especially in high-risk groups, such as male with long-term alcohol drinking.

**Trial registration:**

The registration number on PROSPERO is CRD42021269365.

**Supplementary Information:**

The online version contains supplementary material available at 10.1186/s12889-023-15645-4.

## Background

Alcohol-related liver disease (ALD) is a chronic liver disease related to long-term excessive drinking. ALD is often manifested as alcohol-related fatty liver in the early stage, which may progress to alcohol-related hepatitis, liver fibrosis, cirrhosis, and even liver cancer [1]. Among the annual two million deaths related to liver disease, 50% die from ALD [2]. According to epidemiological data, alcohol intake (volume or history) is closely associated with the risk of liver damage [3].

As reported by the World Health Organization (WHO) [2], the annual per capita alcohol consumption in China is 7.2 L, above the global level (6.4 L) and still on the rise (estimated to reach 8.1 L by 2025 for Chinese people aged 15 and above). Regional epidemiological surveys have shown an increase in the proportion of alcoholics in the general Chinese population, with the prevalence of ALD ranging from 0.50% to 8.55% in some provinces [2, 4–6]. The proportion of ALD in hospitalized patients with liver disease has doubled from 2002 to 2013 [7]. ALD has brought serious health hazards and economic burdens to individuals, families and society.

In China, ALD is diagnosed and treated according to the guidelines established by the Chinese Society of Hepatology [2]. Alcohol abstinence and nutritional supplementation are cornerstones in ALD management, sometimes supported by pharmacological interventions [2, 8, 9]. However, the efficacy and safety of some medications need further evaluation. Critical cases even need liver transplantation (LT). Considering the challenges in treating ALD, early prevention should be carried out based on a full understanding of its epidemiological characteristics.

In China, the prevalence of ALD has been analyzed at the regional level, but never at the national level [10]. Therefore, in this study, we meta-analyzed the epidemiological characteristics of ALD in China.

## Methods

This meta-analysis was performed conforming to the Preferred Reporting Items for Systematic Reviews and Meta-Analyses (PRISMA) guidelines [11], and pre-registered on the International Prospective Register of Systematic Reviews (PROSPERO) with a registration number of CRD42021269365.

### Search strategy

We searched PubMed, Web of Science, Embase, Chinese National Knowledge Infrastructure (CNKI), Chinese Wanfang, and Chinese Biomedicine Literature Database (CBM-SinoMed) for articles published from January 2000 to January 2023 and written in English or Chinese. The keywords included “alcoholic liver disease”, “alcohol liver disease”, “alcohol-related liver disease”, “alcoholic hepatitis”, “alcoholic cirrhosis”, “prevalence”, “epidemiology”, “China”, “Chinese mainland”, “Hong Kong”, “Taiwan”, and “Macao”. The searching strategy is described in Supplement A. We also examined the references in the included studies to enrich the data.

### Inclusion and exclusion criteria

All observational studies were selected if they fulfilled all of the following inclusion criteria: (1) being original articles; (2) including Chinese populations; (3) providing original data on the prevalence of ALD; (4) describing the diagnostic criteria for ALD; (5) existing in a full text; (6) based on a general population rather than patients from one hospital. When duplicate studies were carried out based on the same population, the one with the richest data was selected. Studies were excluded if they: (1) were not published in English or Chinese; (2) were published at least 20 years before; (3) focused on specific groups, such as elderly people, teenagers, or a certain occupation.

After removing the duplicates, two researchers reviewed the titles, abstracts, and full texts of selected articles independently to determine those to be included. Any discrepancy between them was resolved by discussion.

### Quality assessment

Two reviewers independently checked the risk of bias in included studies according to The Joanna Briggs Institute Prevalence Critical Appraisal Tool [12]. The possible bias was evaluated in nine domains, including sample frame, sampling method, sample size, study subjects and setting, data analysis, etc. The details can be found in Supplement B. Each item was rated as “yes”, “no”, “unclear” or “not applicable” according to information available in each study, with a maximum score of nine points. A third reviewer solved disagreements regarding the quality of studies between the two reviewers.

### Data extraction

Data were extracted independently by two researchers. A consensus was reached for any discrepancy through discussion. We used a pre-determined data collection form to extract and record data in excel spreadsheets. The data were about author (s), time of publication, region, residence (urban/rural/mixed), time of study, sampling strategy, sample size, cases, age range, and sex ratio. For articles without providing the time of study, we replaced it with a date subtracting the time of publication by 2 years, based on the average interval between the dates of investigation and publication.

### Statistical analysis

We utilized OpenMeta (Analyst) software and Stata (version 15.0; StataCorp, College Station, TX) to perform meta-analysis. Pooled prevalence of ALD and 95% confidence interval (95% CI) were considered as the measures of effect. Due to the small prevalence of ALD in each study (0 < *P* < 0.2), the data were subjected to double arcsine transformation. Heterogeneity of the eligible studies was evaluated according to Cochrane Q and *I*^*2*^ statistics. A *p*-value less than 0.1 indicated heterogeneity, and *I*^*2*^ values of 25%, 50%, and 75% were considered to represent low, moderate, and high heterogeneity, respectively. A DerSimonian-Laird’s random-effects model was used for studies with significant heterogeneity; otherwise, a fixed-effects model was conducted. A sensitivity analysis was performed to remove studies at a high risk of bias. To detect the source of heterogeneity, subgroup analysis was conducted on sex, region, drinking duration and study year. The region was defined according to the proposal of the National Bureau of Statistics of China: (1) Northeastern China covers Liaoning, Jilin and Heilongjiang; (2) Eastern China covers Beijing, Tianjin, Shanghai, Hebei, Shandong, Jiangsu, Zhejiang, Fujian, Guangdong, Hainan, Taiwan, Hong Kong and Macau; (3) Western China covers Shaanxi, Sichuan, Yunnan, Guizhou, Guangxi Zhuang Autonomous Region, Gansu, Qinghai, Ningxia Hui Autonomous Region, Tibet Autonomous Region, Xinjiang Uygur Autonomous Region, Inner Mongolia Autonomous Region and Chongqing; (4) Central China covers Shanxi, Henan, Anhui, Hubei, Jiangxi and Hunan. We first used a funnel chart to visualize the bias, then Egger’s test and Begg’s test to evaluate the significance. The statistical significance was set at *P* = 0.05.

## Results

### Characteristics of studies selected

**Fig. 1:**
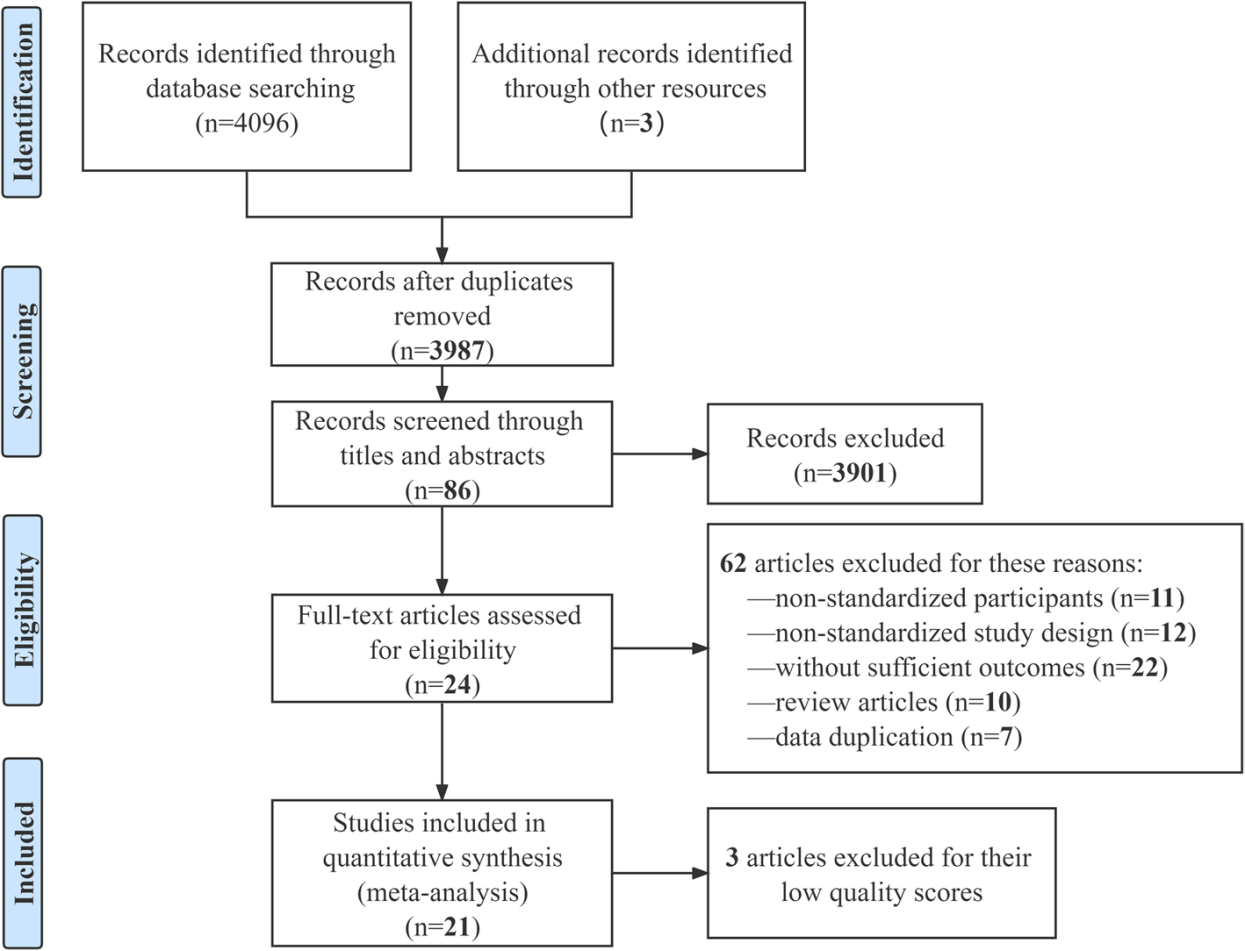
Study flow diagram

As shown in Fig. 1, we initially obtained 4099 results, and 112 were excluded as duplicates. After screening the abstracts and titles in these publications, we retained 86 articles. Then, 62 of them were excluded for incomplete texts, 3 for low-quality. Finally, 21 articles were included, including 6 articles published in English and 15 articles in Chinese.

Their characteristics are summarized in Table 1. The 21 articles were published between 2001 and 2022, of which 7 (33.3%) were published before 2010; 18 articles were conducted in urban–rural settings, 2 in urban regions and 1 in rural regions. In addition, we observed that the prevalence of ALD in both men and women was reported in 13 included articles. Moreover, the populations in these 21 articles were selected from 16 provinces in China. Given this wide coverage, we conducted a subgroup analysis according to four regions introduced by the National Bureau of Statistics of China. The quality of all included articles met the research requirement. The quality assessment is presented in Supplement B.

**Table 1:** Characteristics of the included studies

Studies	Year of Published	Region^a^	Setting	Study year	Sampling strategy	Cases	Sample size	Age range	Male / female^b^
Weixing [13*]	2001	Zhejiang	mixed	1999	stratified sampling	350	4084	≥ 18	7.386
Jinglian, et al. [14*]	2003	Heilongjiang	rural	2001	random sampling	58	1203	NA	1.857
Shengqi, et al. [15*]	2003	Henan	mixed	2000–2003	random sampling	211	4358	NA	1.858
Youming, et al. [16*]	2003	Zhejiang	mixed	NA	multistage stratified cluster sampling	792	18,237	NA	1.944
Xiaolan, et al. [17*]	2004	Shaanxi	mixed	NA	random cluster sampling	83	4115	NA	NA
Shuiqi, et al. [18*]	2005	Hunan	mixed	NA	multistage random stratified cluster sampling	811	18,618	NA	NA
Zhou et al. [19*]	2007	Guangdong	mixed	2005	multistage random stratified cluster sampling	79	3543	7–100	0.587
Jie [20*]	2008	Jilin	mixed	2007	multistage stratified sampling	152	3815	≥ 18	1.042
Lei, et al. [21*]	2010	Guizhou	mixed	2009–2010	NA	186	4167	≥ 18	1.530
Shilin, et al. [22*]	2010	Liaoning	mixed	2007	random cluster sampling	450	6598	7–94	1.642
Jinghui, et al. [23*]	2011	Yunnan	mixed	2010–2011	random cluster sampling	84	1690	≥ 18	NA
Xiaodong, et al. [24*]	2011	Jilin	mixed	2007	multistage stratified cluster sampling	143	3815	≥ 18	0.890
Yan, et al. [25*]	2013	Beijing	mixed	NA	multistage random stratified cluster sampling	309	3762	20–92	1.623
Wang, et al. [26*]	2014	Shandong	mixed	2011	multistage random cluster sampling	624	7295	≥ 18	0.990
Yan, et al. [27*]	2015	Shaanxi, Gansu, Xinjiang	urban	NA	multistage stratified cluster sampling	201	2300	≥ 18	3.007
Huan, et al. [28*]	2016	Shandong	urban	2013–2014	multistage stratified cluster sampling	219	3998	> 20	0.968
Baima KZ, et al. [29*]	2016	Tibet	mixed	2009–2010	random stratified sampling	106	2178	≥ 18	0.829
Qiannan, et al. [30*]	2017	Shaanxi	mixed	2016	NA	397	6723	≥ 18	3.685
Huang, et al. [31*]	2018	Anhui	mixed	2012	multistage random cluster sampling	101	2545	40–96	0.760
Wang H et al. [32*]	2022	Beijing	mixed	2017–2020	multistage stratified cluster sampling	974	74,988	≥ 25	0.855
Pi, et al. [33*]	2022	Beijing	mixed	2018–2020	multistage stratified cluster sampling	311	8014	25–87	0.962

^a^Region: Provincial-level administrative regions in China, including provinces, municipalities and autonomous regions

^b^Male/female: The ratio of males to females

*NA* not available

### Pooled prevalence and stratified prevalence of ALD in China

**Fig. 2:**
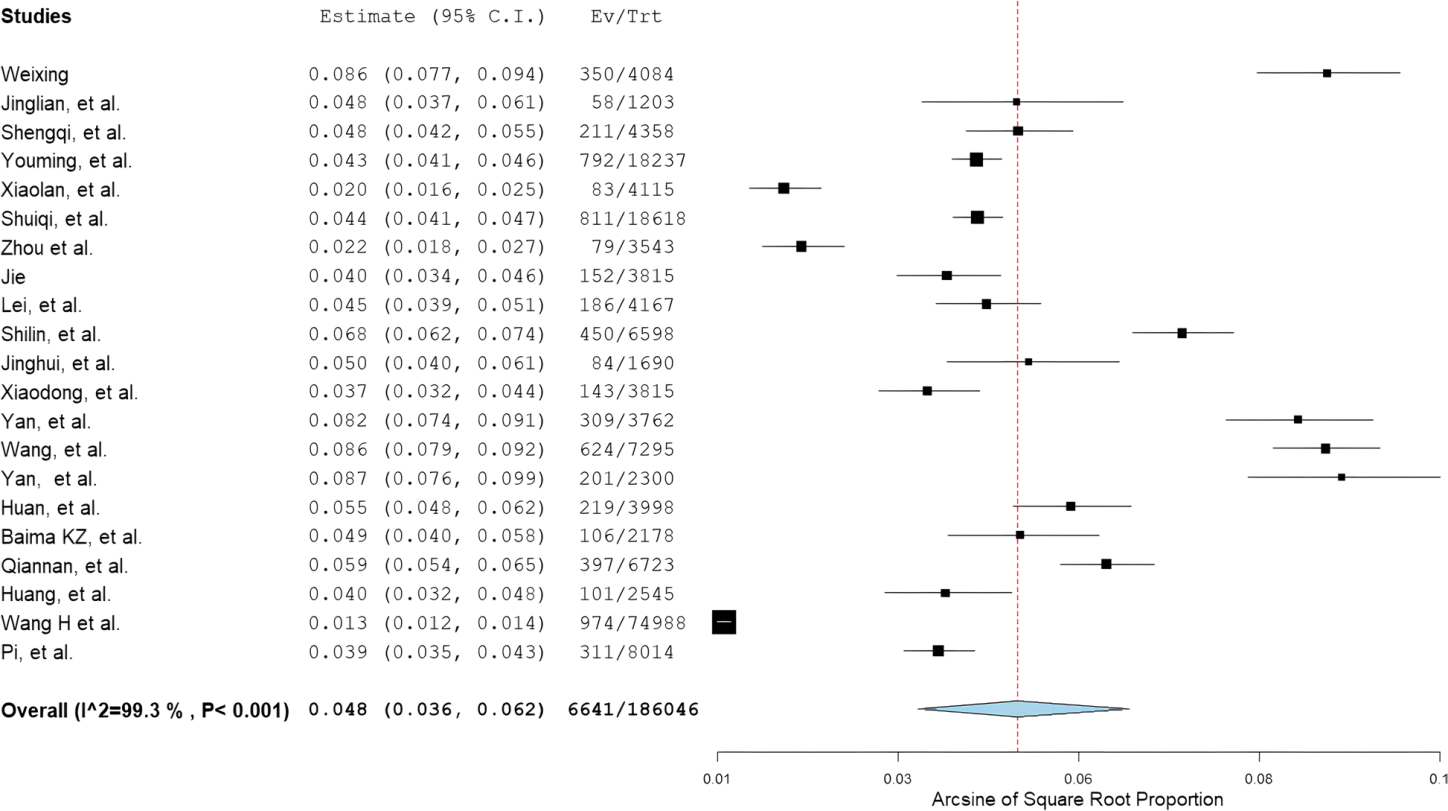
Forest plot of the pooled prevalence of alcohol-related liver disease in China

The pooled prevalence of ALD in China was 4.8% (95% CI, 3.6%-6.2%), as shown in random-effects meta-analysis (Fig. 2).

The prevalence was analyzed in subgroups (Table 2) set according to the following categories: sex (male, female), study region (Northeastern, Eastern, Western, and Central China), drinking duration (below 5 years, 5–10 years, above 10 years), and time of study (1999–2004, 2005–2010, 2011–2016, 2017–2022). The higher prevalence of ALD was observed in men, West China, and people with more than 10 years of drinking history. Sex was closely associated with ALD prevalence (9.3% [95% CI, 4.4%-16.0%] in men vs. 2.0% [95% CI, 0.0%-6.7%] in women). The prevalence of ALD did not show a trend related to study time: 4.7% (95% CI, 3.0%-6.7%) in 1999–2004, 4.3% (95% CI, 3.5%-5.3%) in 2005–2010, 6.7% (95% CI, 5.3%-8.3%) in 2011–2016, and 2.4% in 2017–2022.

**Table 2:** Stratified prevalence of alcohol-related liver disease in China

Subgroups	Prevalence (%) (95% CI)	Number of studies	Heterogeneity		Case/total
			*I*^2^ (%)	*P* value	
Sex					
Male	9.3 (4.4, 16.0)	13	99.86	< 0.001	8111/82165
Female	2.0 (0.0, 6.7)	13	99.87	< 0.001	2752/71280
Location^a^					
Northeastern	4.8 (3.3, 6.5)	4	95.22	< 0.001	803/15431
Eastern	4.9 (2.7, 7.7)	8	99.66	< 0.001	3658/123921
Western	5.0 (3.3, 6.9)	6	97.16	< 0.001	1057/21173
Central	4.4 (4.0, 4.8)	3	37.46	0.202	1123/25521
Drinking duration (years)					
< 5	0.9 (0.2, 1.9)	7	92.77	< 0.001	76/14635
5–10	4.6 (3.0, 6.5)	9	88.07	< 0.001	203/4966
≥ 10	9.9 (6.5, 14.0)	9	98.64	< 0.001	2059/19620
Study year					
1999–2004	4.7 (3.0, 6.7)	5	97.99	< 0.001	1494/31997
2005–2010	4.3 (3.5, 5.3)	8	94.82	< 0.001	2011/44424
2011–2016	6.7 (5.3, 8.3)	6	95.71	< 0.001	1851/26623
2017–2022	2.4 (0.5, 5.6)	2	99.51	< 0.001	1285/83002
Total	4.8 (3.6, 6.2)	21	99.30	< 0.001	6641/186046

^a^Location: according to the proposal of the National Bureau of Statistics of China

Northeastern China covers Liaoning, Jilin and Heilongjiang

Eastern China covers Beijing, Tianjin, Shanghai, Hebei, Shandong, Jiangsu, Zhejiang, Fujian, Guangdong, Hainan, Taiwan, Hong Kong and Macau

Western China covers Shaanxi, Sichuan, Yunnan, Guizhou, Guangxi Zhuang Autonomous Region, Gansu, Qinghai, Ningxia Hui Autonomous Region, Tibet Autonomous Region, Xinjiang Uygur Autonomous Region, Inner Mongolia Autonomous Region and Chongqing

Central China covers Shanxi, Henan, Anhui, Hubei, Jiangxi and Hunan

**Fig. 3:**
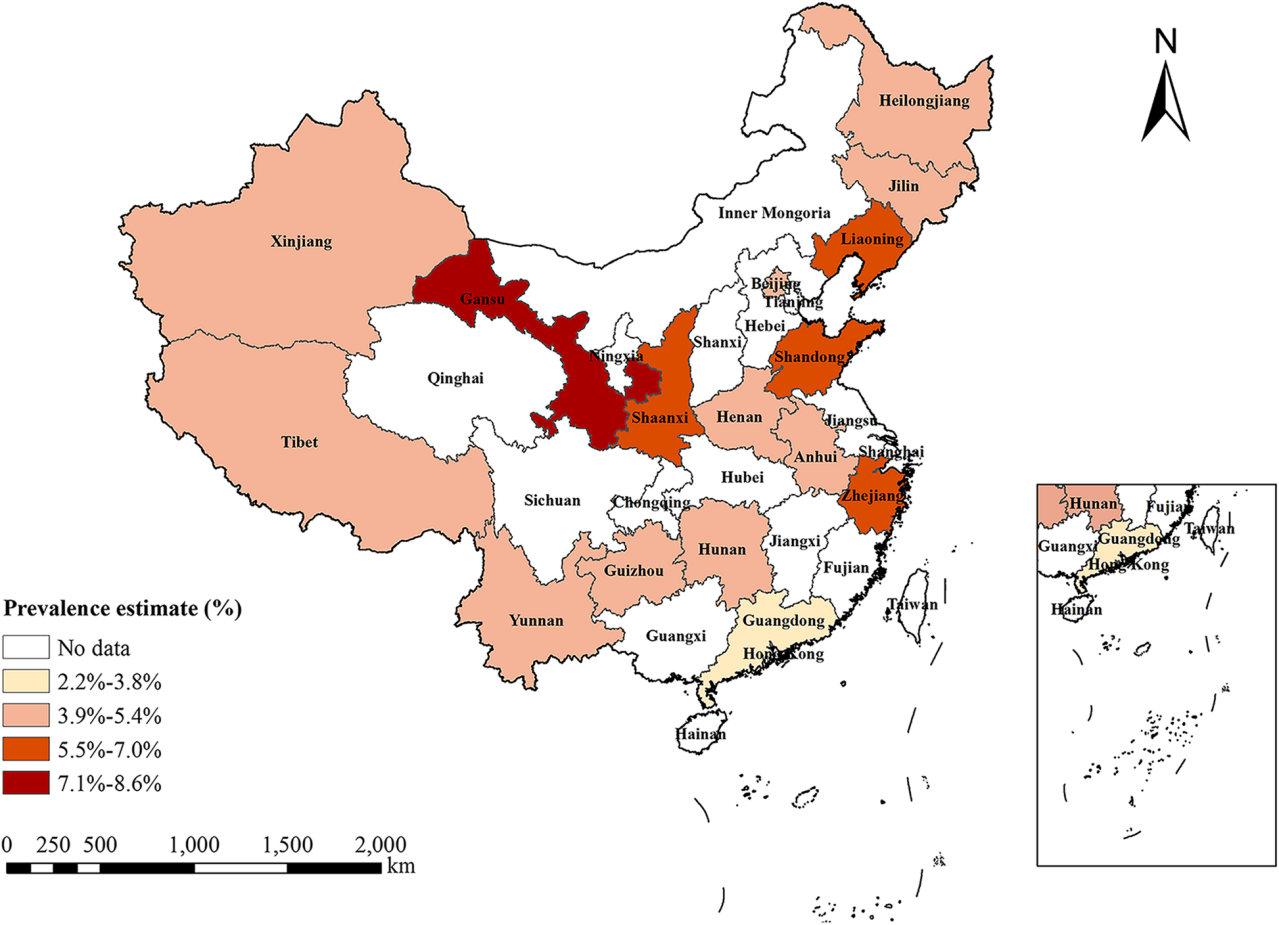
Regional distribution of pooled prevalence of alcohol-related liver disease in China

The prevalence of ALD varied evidently across four Chinese geographical regions (Table 2). The prevalence of ALD was the highest (5.0% [95% CI, 3.3%-6.9%]) in West China and the lowest (4.4% [95% CI, 4.0%-4.8%]) in Central China. The prevalence in East China was similar (5.0% [95% CI, 3.3%-6.9%]) to that in West China, while higher than that in Northeast China (4.8% [95% CI, 3.3%-6.5%]). More evident regional difference in ALD prevalence was seen between provinces of China (Fig. 3).

Also noticed in Table 2, the prevalence of ALD was higher in the subgroup with a longer drinking history than in the subgroup with a shorter drinking history. The prevalence of ALD in the subgroups with a drinking history of less than 5 years, 5–10 years, and more than 10 years was 0.9% (95% CI, 0.2%-1.9%), 4.6% (95% CI, 3.0%-6.5%), and 9.9% (95% CI, 6.5%-14.0%), respectively.

### Analysis of heterogeneity and publication bias

Significant overall heterogeneity was noted in the included studies (*P* < 0.001, *I*^2^ = 99.30%). Therefore, we conducted a subgroup analysis, and unfortunately observed that the heterogeneity was not reduced in most subgroups. The sensitivity analysis showed that the pooled prevalence of ALD varied from 4.7% (95% CI, 3.5%-6.0%) to 5.1% (95% CI, 4.3%-5.9%) as each study was serially excluded, and no single study had a substantial influence on the pooled prevalence (Supplement C). Despite the apparent asymmetry of the funnel plot (Supplement D), no significant publication bias was identified by the Begg’s test (*P* = 0.365) with double arcsine transformation. However, Egger’s test revealed a significant publication bias (*P* < 0.001) (Supplement D).

## Discussion

This systematic review and meta-analysis comprehensively described the prevalence of ALD in China based on data published from 2001 to 2022. We analyzed 21 epidemiological surveys covering 16 provincial regions in China. The pooled prevalence of ALD in China was 4.8% (95%CI, 3.6%-6.2%), which is consistent with that reported by authoritative institutions (range 2.3% to 6.1%, median 4.5%) [4]. Previous studies have shown that the prevalence in European countries was estimated at 6%, which is comparable to our finding [5, 34]. Furthermore, the prevalence of ALD in China is obviously higher than that in Japan (1.56% to 2.34%) [34] and South Korea (about 1.7%) [35].

Alcohol consumption pattern has undergone continuous change around the world [36]. China has become a country with the highest per-capita consumption of pure alcohol [5]. The proportion of regular drinkers among Chinese adults rose from 27.0% in 2000 to 66.2% in 2015, and the proportion of heavy drinkers rose from 0.21% in 1982 to 14.8% in 2000 [5]. In the present study, the prevalence of ALD in 2011–2016 (6.7%) was higher than that in 1999–2004 (4.7%) and 2005–2010 (4.3%), with an upward trend similar to that of proportion of drinkers. Interestingly, the ALD prevalence in 2017–2022 was only 2.4%, which was markedly lower than that in other subgroups. The combined prevalence in this subgroup may be associated with the small number of included studies. Besides, this result cannot be explained by the rise of alcohol desire and consumption in the Chinese population during the COVID-19 epidemic [37–39]. Studies have shown that during the COVID-19 epidemic, some individuals relieve mental stress and negative emotions through drinking alcohol, which may lead to or exacerbate problems such as alcohol abuse or dependence, further impairing their physical and mental health [40, 41]. Therefore, the impact of the epidemic on the prevalence of ALD deserves concern. However, investigations of ALD in China during the epidemic are still sporadic. It is important to note that two studies included in this subgroup were conducted in 2017–2020 and 2018–2020, and the detailed data in different years were not provided, so it is not rigorous to equate this prevalence to that of ALD during the COVID-19 epidemic. Considering the delay in the occurrence of ALD induced by drinking behavior [1], the impact of epidemic on ALD cannot be reflected by its prevalence. In the future, we hope that more studies on the epidemiological characteristics of ALD patients in the post-epidemic era will fill this gap. Meanwhile, previous studies have shown that drinking history is a risk factor for ALD [6, 42], which is supported by our research results: the prevalence of those with a drinking history of more than 10 years was notably higher than those with a drinking history of less than 5 years.

The prevalence of ALD varied prominently across geological regions in China. The pooled prevalence in West China was 5.0%, higher than the overall prevalence and that in Central China. There is a lack of studies on the association between geographical region and ALD in China, but a recent study [43] has pointed out that liver function indicators, alanine aminotransferase (ALT), is related to geographical factors. In the above study, the ALT level showed a spatial trend: high in the west and low in the east; high in the north and low in the south. Considering that ALD can increase laboratory ALT [44], we speculate that the prevalence of ALD may be influenced by geographical factors, but this requires the verification in more studies. Besides, the alcohol drinking habits across China probably also contribute to the region-related difference in ALD prevalence.

Another major finding of this study is that the prevalence of ALD was remarkably different between men (9.3%) and women (2.0%) in China. Numerous studies [45–49] have shown that men drink much more alcohol than women in China and many other countries [35, 50, 51], which can explain the sex-related difference in ALD prevalence. Actually, the alcohol consumption and ALD prevalence have both increased among women in recent years. As reported by the WHO, the proportion of female drinkers in the Western Pacific Region has gradually increased from 39.3% in 2000 to 40.7% in 2016, with an obvious upward trend [46]. Besides, a study [52] based on the Global Burden of Disease database states that from 2000 to 2017, the sex-related difference in ALD prevalence has become less prominent in the Nordic countries. Few studies have been conducted to analyze alcohol consumption and alcohol-related liver disease in Chinese women; thus, the gender-related trends in ALD prevalence over time should be further explored.

To date, this is the first meta-analysis to describe the total prevalence of ALD in China. Besides, we strictly controlled the quality of included articles. Moreover, double arcsine transformation of data was conducted before implementing robust statistical methods, thereby improving the reliability of the results [53].

Several limitations also exist. First, the results of the study are highly heterogeneous, limited by the nature of the meta-analysis of single-group rates. We had already attempted to avoid the potential publication bias in study screening, quality evaluation and data processing. Sensitivity analysis allowed us to obtain more stable results. We also adopted methods such as stratified analysis to reduce study heterogeneity. After subgroup analysis, the heterogeneity remains unexplained. In this study, various diagnostic methods for ALD may bring about high heterogeneity between included studies. Even if statistical heterogeneity may be excluded, the heterogeneity between clinical research cannot be eliminated. Second, we only collected data from some provinces of China, which may have a potential impact on the representativeness of the results. Third, most of included studies were carried out before 2010, and recent epidemiological studies lack.

## Conclusions

This is the first meta-analysis to explore the total prevalence of ALD in China. The prevalence of ALD is relatively high and has increased slightly in China over the past few decades. More research is needed in the future to validate our results from this study.

## Supplementary Information

**Additional file 1:** **Supplement A.** Search strategies of different databases. **Supplement B.** The quality assessment for the included studies. **Supplement C.** Forest plot of sensitivity analysis. **Supplement D.** The publication bias for the included studies.

## Data Availability

All datasets generated and/or analyzed during the current research are included in this article and its supplementary information file.

## Abbreviations

ALD: Alcohol-related liver disease
CNKI: Chinese National Knowledge Infrastructure
CBM-SinoMed: Chinese Biomedicine Literature Database
CI: Confidence interval
WHO: World Health Organization
LT: Liver transplantation
PRISMA: Preferred Reporting Items for Systematic Reviews and Meta-analyses
ALT: Alanine aminotransferase
