# Supplementary material for: Epidemiological characteristics of alcohol-related liver disease in China: a systematic review and meta-analysis
Source: BMC Public Health. 2023 Jul 1;23:1276. doi: 10.1186/s12889-023-15645-4 (PMC10314568; doi:10.1186/s12889-023-15645-4)
Supplement: Supplementary file 1 — Additional file 1: Supplement A. Search strategies of different databases. Supplement B. The quality assessment for the included studies. Supplement C. Forest plot of sensitivity analysis. Supplement D. The publication bias for the included studies. [file 12889_2023_15645_MOESM1_ESM.docx]

Supplementary Content

[Supplement A. Search strategies of different databases 2](#_Toc127219544)

[Supplement B. The quality assessment for the included studies 3](#_Toc127219545)

[Supplement C. Forest plot of sensitivity analysis 4](#_Toc127219546)

[Supplement D. The publication bias for the included studies 5](#_Toc127219547)

## Supplement A. Search strategies of different databases

| **Database** | **search terms** |
| --- | --- |
| PubMed | (("alcoholic liver disease" OR "alcohol liver disease" OR "ALD" OR "alcohol-related liver disease") AND (prevalence [Title/Abstract] OR epidemiology [Title/Abstract])) AND (China [Title/Abstract] OR mainland China [Title/Abstract] OR Hong Kong [Title/Abstract] OR Taiwan [Title/Abstract] OR Macao [Title/Abstract]) |
| Embase | 1. (alcohol liver disease or alcoholic liver disease or alcohol-related liver disease or ALD).ab.  2. (alcohol liver disease or alcoholic liver disease or alcohol-related liver disease or ALD).ti.  3. (prevalence or epidemiology).ab.  4. (prevalence or epidemiology).ti.  5. (China or mainland China or Hong Kong or Taiwan or Macao).af.  6. 1 or 2  7. 3 or 4  8. 5 and 6 and 7. |
| Web of Science | ((TS= (alcohol liver disease OR alcoholic liver disease OR alcohol-related liver disease OR ALD)) AND TS= (prevalence OR epidemiology)) AND AD= (China OR mainland China OR Hong Kong OR Taiwan OR Macao). |
| CNKI^†^ | FT = (‘alcohol-related liver disease’+ ‘alcohol-related liver injury’+ ‘alcohol-related hepatitis’+ ‘alcohol-related liver cirrhosis’) AND SU = (‘prevalence’+ ‘epidemiology’+ ‘epidemic’)  FT=full text；SU=subject |
| Chinese Wanfang | all:("alcohol-related liver disease" or "alcohol-related liver injury" or "alcohol-related hepatitis" or "alcohol-related liver cirrhosis") and all:("prevalence" or "epidemiology" or "epidemic"). |
| CBM-SinoMed^‡^ | ("alcohol-related liver disease"[core fields] OR "alcohol-related liver injury"[core fields] OR "alcohol-related hepatitis"[core field] OR "alcohol-related liver cirrhosis"[core field]) AND( "prevalence"[common field] OR "epidemiology"[common field] OR "epidemic"[common field]). |
| † Chinese National Knowledge Infrastructure.  ‡ Chinese Biomedicine Literature Database.  Note: CNKI, Chinese Wanfang and CBM-SinoMed are searched in Chinese language. This table is displayed in English language for reading convenience. | |

## Supplement B. The quality assessment for the included studies


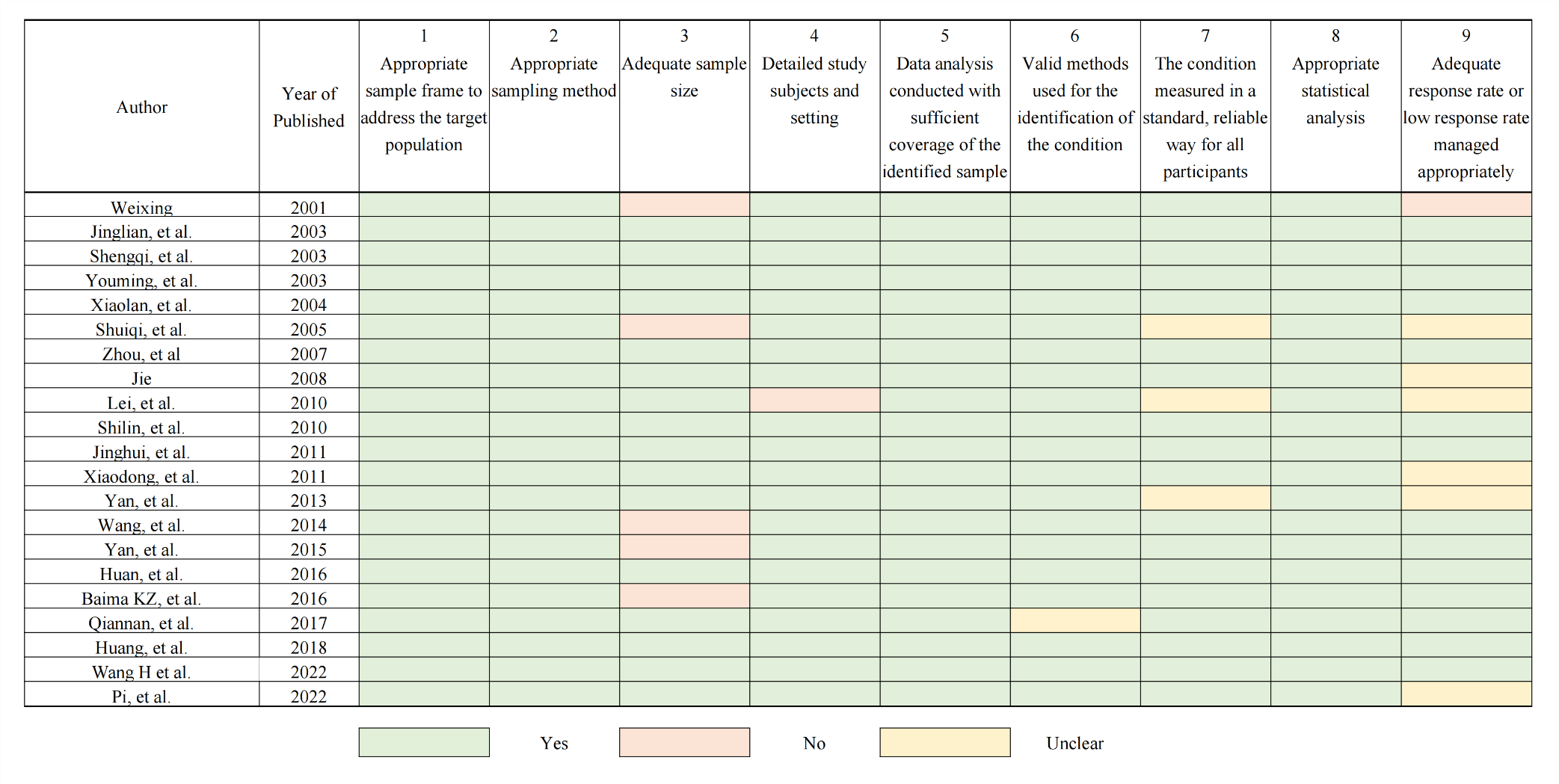
Figure B The quality assessment for the included studies

## Supplement C. Forest plot of sensitivity analysis


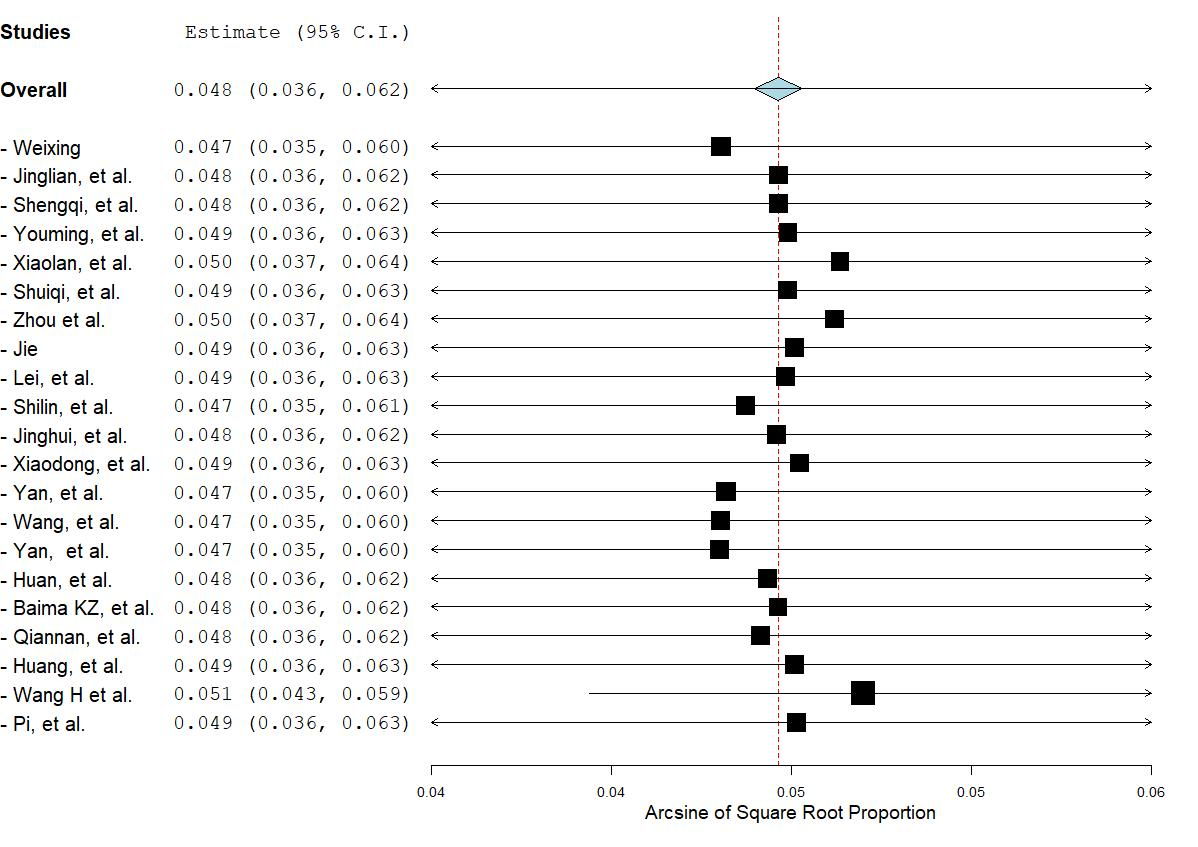


Figure C. Forest plot of sensitivity analysis

## Supplement D. The publication bias for the included studies


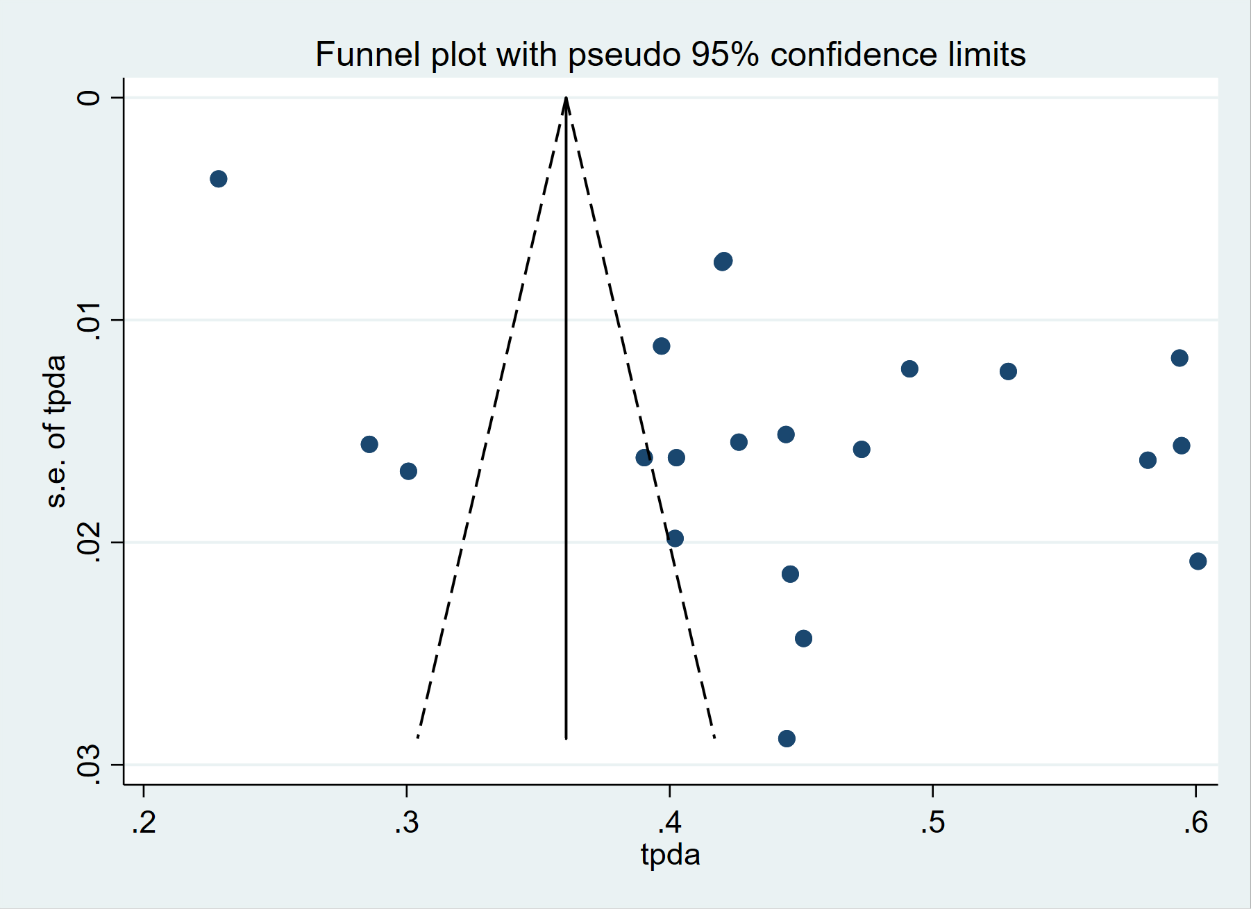


Figure D.1 Funnel plot for publication bias


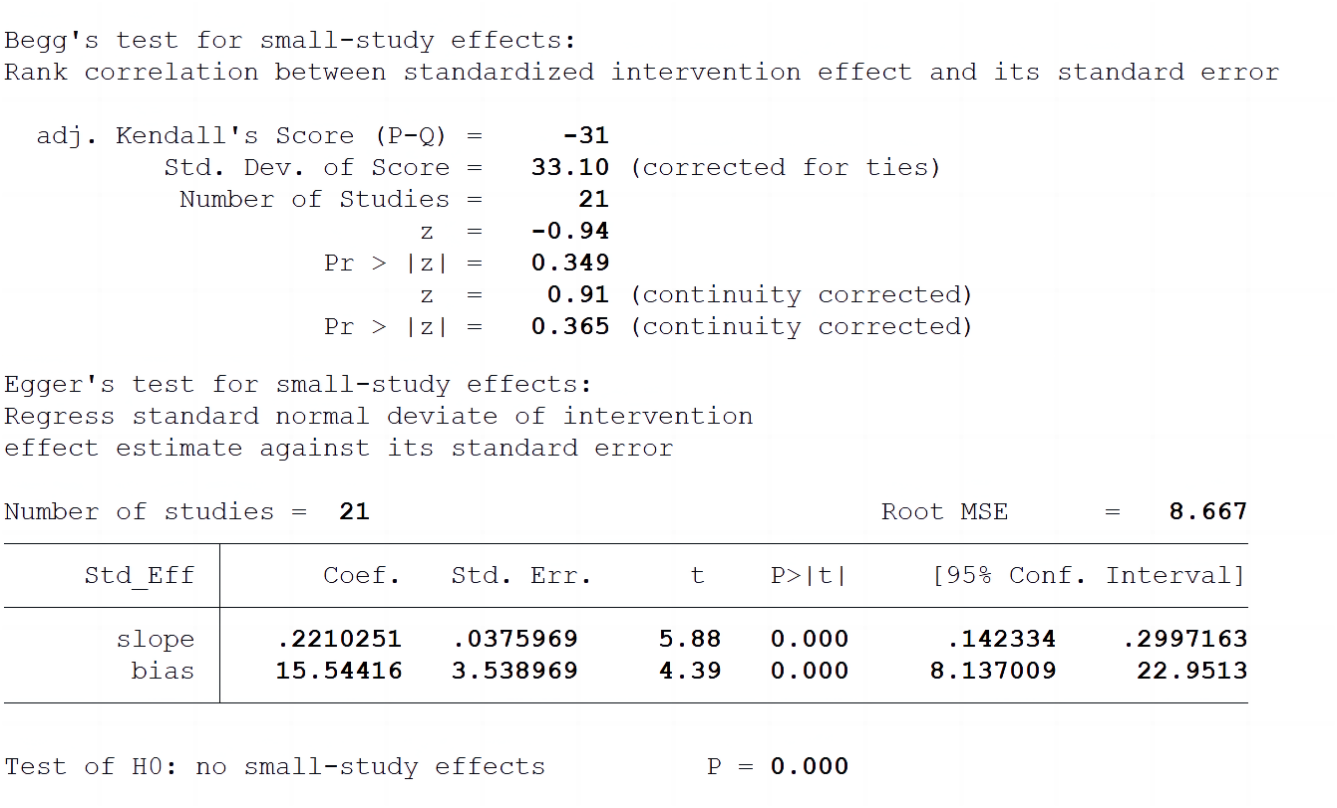


Figure D.2 Begg’s test and Egger’s test for publication bias
